# Microbial Adaptation Due to Gastric Bypass Surgery: The Nutritional Impact

**DOI:** 10.3390/nu12041199

**Published:** 2020-04-24

**Authors:** Silke Crommen, Alma Mattes, Marie-Christine Simon

**Affiliations:** 1 Department of Nutrition and Food Sciences, Nutritional Physiology, University of Bonn, 53115 Bonn, Germany; 2 Department of Nutrition and Food Sciences, Nutrition and Microbiota, University of Bonn, 53115 Bonn, Germany

**Keywords:** bariatric surgery, Roux-en-Y gastric bypass, gastric bypass surgery, gut microbiome, microbiota, diet, obesity, weight loss

## Abstract

Bariatric surgery leads to sustained weight loss and the resolution of obesity-related comorbidities. Recent studies have suggested that changes in gut microbiota are associated with the weight loss induced by bariatric surgery. Several studies have observed major changes in the microbial composition following gastric bypass surgery. However, there are inconsistencies between the reported alterations in microbial compositions in different studies. Furthermore, it is well established that diet is an important factor shaping the composition and function of intestinal microbiota. However, most studies on gastric bypass have not assessed the impact of dietary intake on the microbiome composition in general, let alone the impact of restrictive diets prior to bariatric surgery, which are recommended for reducing liver fat content and size. Thus, the relative impact of bariatric surgery on weight loss and gut microbiota remains unclear. Therefore, this review aims to provide a deeper understanding of the current knowledge of the changes in intestinal microbiota induced by bariatric surgery considering pre-surgical nutritional changes.

## 1. Introduction

**Figure 1:**
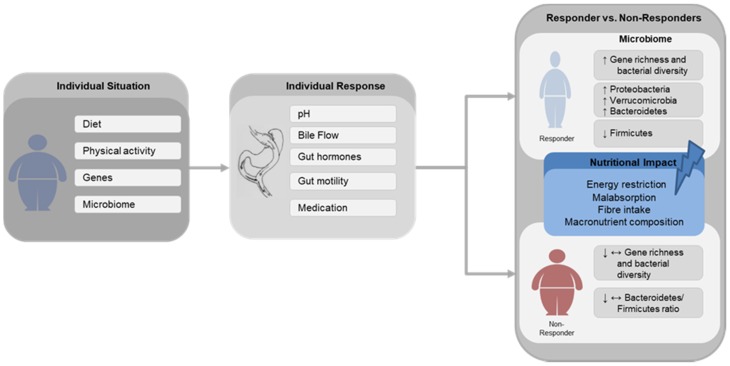
Schematic Illustration Schematic illustration of the main gut microbial changes associated with successful (Responder) and poor (Non-Responder) weight loss after gastric bypass surgery and the possible impact of nutritional factors. Diet, physical activity, genes and gut microbiome composition are widely described factors leading to obesity. Following gastric bypass surgery, the individual response is affected by alterations in pH, bile flow, changes in gut hormones secretion, gut motility and medication usage. ↑—increase, ↓—decrease, ↔—unchanged.

The increasing prevalence of obesity, caused by the changing dietary and exercise habits, seems to have reached epidemic proportions worldwide with more than 650 million adults being affected in 2016 [1]. Western diets, defined by a high fat and low fibre intake, sedentary lifestyle and genetics, are common causes of obesity [2]. Recent findings have suggested that gut microbiota play a role in the onset of obesity by contributing to energy homeostasis and fat storage [3,4,5] (Figure 1). Furthermore, there is evidence that gut microbiota varies in lean and obese individuals [4,6,7]. In particular, there is a difference in the intestinal ratio of Bacteroides and Firmicutes between lean and obese individuals with a greater relative abundance of Firmicutes in obese individuals. At present, only bariatric surgery seems to induce sustained weight loss and resolution of obesity-related morbidities, such as type 2 diabetes mellitus (T2DM), non-alcoholic fatty liver disease (NAFLD), hypertension and cardiovascular disease [8,9,10,11,12,13], relatively attributable to microbial alterations.

Diet is an important factor shaping the composition and function of intestinal microbiota. However, most studies on gastric bypass have not assessed the impact of dietary intake in general. Additionally, the effects of restrictive diets prior to bariatric surgery, which are recommended for reducing liver fat content and size, on the microbiome composition were not investigated in detail. Thus, the relative impact of bariatric surgery on weight loss and gut microbiota remains unclear. Therefore, this review aims to provide a deeper understanding of the changes in intestinal microbiota induced by bariatric surgery considering pre-surgical nutritional changes.

## 2. Materials and Methods

A systematic literature search was performed on PubMed using the search terms “gastrointestinal microbiome”, “gastrointestinal microbiota”, “microbiome”, “microbiota”, “gut microbiome”, “bariatric surgery”, “gastric bypass”, “Roux-en-Y gastric bypass”, RYGB”, “mini gastric bypass” and “MGB” individually or in combination. We selected publications between February 2009 and January 2020 containing original research on humans. Of the selected articles, the full texts, as well as the references, were reviewed. If the reference list contained eligible articles, those were also included. All publications not composed in the English language were excluded.

## 3. The Intestinal Microbiome in Obesity

Obesity is associated with changes in the relative abundance of the two dominant bacterial divisions: Bacteroidetes and Firmicutes [4]. Ley et al. discovered in a study comparing lean and obese mice that the ob/ob animals showed a 50% reduction in the abundance of Bacteroidetes, whereas the level of Firmicutes was higher by a corresponding degree [6]. Analogous differences can be observed in the distal gut microbiota of obese versus lean humans [4]. Animal models have produced evidence for the causal role of intestinal microbiota in the aetiology of obesity and insulin resistance [14]. Turnbaugh et al. showed that faecal microbial transplantation (FMT) of faeces from obese mice into lean, germ-free mice (GF) led to a marked increase in body weight of the recipient animals [4]. The obese phenotype seems to be transmissible and is promoted by microbiota with an increased capacity to harvest energy from the host’s diet [4,15]. In line with these findings, a study where faecal microbiota from a pair of twins, discordant in their obesity status, was transplanted into GF mice showed that recipients from the obese donor gained significantly more weight than their counterparts with the lean donor [16]. Such causal relation is scarce for humans; however, one case report depicts a woman successfully treated with FMT who developed new-onset obesity after receiving stool from a healthy but overweight donor [17]. Regarding the improvement of obesity-associated metabolic parameters, Zhang et al. found mixed results, as two of the reviewed studies reported an improved peripheral insulin sensitivity after FMT, while some other studies showed no differences in fasting plasma glucose, hepatic insulin sensitivity or BMI after following FMT [18].

The variance of human gut microbiota composition and clinical phenotypes is huge. Based on the Flemish gut flora project and Lifelines DEEP (LLD) cohort, a human core microbiota could be identified. It is expected to be representative of the average gut microbiota composition in the Western European population. While 664 genera were identified, total microbial richness still seems to be underexplored [19]. Age and gender of the study population did not only correlate with microbial composition distance and diversity but also with functional richness [20]. An association between microbiome composition and BMI was small but significant [19]. In the LLD cohort, obese-specific microbial associations were found for lipid compositions in the VLDL and LDL lipoprotein subclasses. In obese individuals, bacterial L-methionine biosynthesis and a Ruminococcus species were associated with cardiovascular phenotypes (i.e., atherosclerosis and liver fat content) [21].

Obese individuals show an altered intestinal ratio of Bacteroidetes and Firmicutes with a greater relative abundance of Firmicutes [15]. A reduction in energy intake is able to lower the ratio because the relative abundance of Bacteroidetes increases as obese individuals lose weight on either a fat- or carbohydrate-restricted low-calorie diet [4,15]. Three genera of bacteria are often overrepresented in obese humans, including Bacteroides and Prevotella (both Bacteroidetes) and Ruminococcus (Firmicutes) [22]. In a study by Turnbaugh et al., diet-induced obesity (DIO) produced a bloom in a single uncultured clade within the Mollicutes class of Firmicutes. It became the dominant lineage within distal gut microbiota concurrently accompanied by a division-wide suppression of Bacteroidetes. This finding suggests that the Mollicute lineage has increased fitness relative to other Firmicutes and Bacteroidetes [23].

Bacteria that cause weight gain are thought to induce the expression of genes related to the lipid and carbohydrate metabolism resulting in a greater energy harvest from the diet [24]. Humans get approximately 10% of their daily energy supply from short-chain fatty acids (SCFAs) produced by the gut bacteria. SCFAs act not only as energy substrates for the host but also as signalling molecules thereby influencing energy intake and metabolism [25,26]. However, these SCFA profiles, along with butyrate-producing bacteria, are altered in obese individuals [27].

Using these recent studies, it is not possible to confirm whether associations between obesity and the two dominant bacterial phyla exist because there are discrepancies in the Bacteroidetes/Firmicutes ratio and, therefore, its relation to obesity [24,28]. It is likely that the influence of the gut microbiome on obesity is much more complex than simply an imbalance in the proportion of bacteria phyla [24]. Phylum-wide changes in the gut microbiota cannot be currently considered as biomarkers for obesity [25]. Due to a variety of confounding factors within the human population (heterogeneity in genotype, lifestyle, diet, ethnicity), a suitable definition of an “obese” microbiota is currently impossible [25,29]. In addition, the causal relationship and underlying mechanism remain outstanding [25].

## 4. Impact of Gastric Bypass Surgery on Gut Microbiota

Bariatric surgery is currently the most effective treatment option for achieving sustained weight loss and the resolution of obesity-related comorbidities, such as T2DM, NAFLD, cardiovascular disease and reduced mortality [9,10,11,12,13]. Bariatric surgery is recommended for individuals with a BMI of ≥ 40 kg/m² or a BMI of > 35 kg/m² with obesity-related comorbidities [30]. There are several bariatric surgery procedures, of which Roux-en-Y gastric bypass (RYGB) is one of the most frequently undertaken procedure globally [31,32], due to the profound weight loss [33] and cardiometabolic improvement observed after this surgery [34,35]. RYGB surgery consists of a reduction of gastric volume by forming a small gastric pouch and a section of jejunum is then connected to the pouch. Thereby, the majority of the stomach, duodenum and the proximal jejunum are excluded from the intestinal tract [36].

Bariatric surgery significantly helps to ameliorate biochemical and histologic parameters in patients with NAFLD [37]. Steatosis, Steatohepatitis and liver fibrosis are improved in the majority of patients after surgery [38]. In 84% of the patients, liver function test values were normalised following bariatric surgery by the end of the first postoperative year. Hereby, both RYGB and SG proved to be similarly effective [39]. RYGB and SG surgery also significantly improved alanine aminotransferase, aspartate aminotransferase, NAFLD activity score and NAFLD fibrosis score [37]. Hepatic insulin resistance was markedly decreased post-surgery, while beta cell function improved due to an increase in postprandial GLP-1 level [40,41]. In a study by Feng et al. fasting insulin and 120 min insulin decreased significantly in post-RYGB patients. Decreases in HbA1c and fasting blood glucose levels were also noticed and reached normal levels at 1–3 months after surgery. Complete NAFLD remission was achieved in 96% of post-RYGB patients as well as diabetes remission in 48% of post-RYGB patients, thereby improving cardiovascular risk factors [42].

Generally, initial excess weight loss is about 60%–76% for RYGB patients in the first five years [34,43]. However, the total amount of weight loss shows high inter-individual variability with a large proportion of patients who achieve a large loss of weight (responder) and a subset of patients who fail to achieve the expected weight loss during the first postoperative year or even regain weight afterwards and, therefore, these patients gain little health benefit from the surgery (non-responder) [44,45,46] (Figure 1). Currently, there are several possible reasons being discussed to account for the high inter-individual variability, such as genetic, epigenetics [47], biological and clinical factors [48,49,50]. In particular, age, pre-surgical presence of T2DM, higher initial BMI and behavioural problems are associated with poor weight loss after bariatric surgery [51]. Furthermore, it is likely that differential changes in gut microbiota composition also account for the variability seen after gastric bypass procedures. Furet et al. found that a higher ratio of Bacteroides to Prevotella following RYGB leads to higher weight loss and elevated blood leptin levels. However, these associations were dependent on energy intake indicating that the microbial alterations observed after RYGB might be caused by energy restriction [52]. Conversely, in a recent study conducted by Fouladi et al., no differences were found in the gut microbiome composition between patients after RYGB with successful weight loss (SWL) and poor weight loss (PWL). Thus, a humanised mouse model was used to elucidate the possible differences in the composition and function of the microbiome of SWL individuals, PWL individuals and non-surgical control (NSC) individuals who were matched for age and BMI to the SWL-group. Transplantation of faecal samples from SWL, PWL and NSC patients into antibiotic-treated mice revealed that mice colonised with the PWL microbiome gained more weight than mice transplanted with the SWL microbiome even though food intake did not differ between the two groups. In this study, the genus Barnesiella was associated with weight outcome and showed a higher abundance in the PWL recipient mice compared to the SWL and NSC recipient mice. The authors hypothesised that the microbiota from PWL subjects contribute to the observed weight gain after RYGB surgery independent of food intake. This could be due to an increase in energy absorption from diet and increased fat accumulation in adipose tissue inducing low-grade inflammation and metabolic alterations. Rather than compositional differences, there could be at least some functional differences in the gut microbiome of PWL and SWL patients [53].

Several studies have indicated that RYGB surgery not only changed the microbiota composition but also the microbial functions. Thus, enhanced protein degradation, an increase in functional annotations and the associated fatty acid utilisation are widely observed after RYGB surgery [54,55,56,57]. This led to the assumption that, after gastric bypass surgery, the energy harvest from the diet is decreased.

Further studies have indicated that the restriction and malabsorption induced by the surgery are not the only cause of the observed metabolic improvements. Rather, findings have suggested that changes in the intestinal microbiota exert considerable influence on surgically induced weight loss and metabolic improvement [55,56,58,59,60,61]. Animal studies have shown that RYGB surgery led to a rapid and sustained increase in the abundance of certain microbes, such as Escherichia and Akkermansia, independent of weight loss and energy restriction. After faecal transplantation from RYGB-treated mice to non-operated germ-free mice, recipient mice exhibited a decrease in the rate of weight gain and a decreased mass of body fat [62] suggesting that there could be a direct link between the alterations in the gut microbiome and the weight-reducing effects seen after gastric bypass surgery. In a study conducted by Tremaroli et al. RYGB surgery caused long-lasting effects on the composition and functional capacity of the human gut microbiota. Furthermore, the authors colonized germ-free mice with stool from postoperative patients and also demonstrated that the surgically altered microbiota promoted reduced fat deposition in recipient mice. These findings led to the hypothesis that there could be a causal relationship between the microbial alterations following gastric bypass surgery and the observed weight reduction [56].

Several human studies have shown major changes in the gut microbiota composition at the phylum level after gastric bypass (Table 1). Moreover, in terms of the distinct surgical procedures, different microbiota-related alterations have been reported, while most profound changes in gut microbiota were observed after RYGB surgery compared to sleeve gastrectomy (SG) or adjustable gastric banding [54,59,63,64]. In general, microbial diversity and richness increased within three months of post-RYGB surgery and continued even after one or two years after the operation [54,57,60,65]. Physiological and anatomical modifications induced by RYGB surgery could be responsible for the increase in diversity and microbial richness. This is possibly due to the altered gut environment, particularly an increase in pH and oxygen content, which allows acid-sensitive bacteria and facultative anaerobic bacteria to colonise [66]. As a result of the higher oxygen level, the relative abundance of the aero-tolerant species E. coli was found to increase after RYGB surgery [52,56,57]. On a taxa level, an increase in Bacteroidetes, Proteobacteria and Verrucomicrobia (e.g., Akkermansia muciniphila) and a decrease in members of the phylum Firmicutes have been consistently observed in humans after gastric bypass procedures compared to the same patients preoperatively or obese patients who have not undergone bariatric surgery (Table 1). Zhang et al. were the first to compare the gut microbiome composition of three normal weight, three morbidly obese and three post-RYGB patients. Lower abundance of Firmicutes, Clostridia and Verrucomicrobia were observed in the RYGB patients compared to those of the normal weight and morbidly obese subjects while the abundance of the class Gammaproteobacteria was markedly increased for the RYGB patients. Additionally, on a family taxonomic level, the relative abundance of Enterobacteriaceae, Fusobacteriaceae and Akkermansia were markedly increased in the post-bariatric group [67]. A recent study comparing the gut microbiota adaptation after RYGB or SG surgery showed different gut microbiota profiles between these surgical procedures [63]. SG surgery was associated with higher levels of Akkermansia, Eubacterium, Haemophilus and Blautia, whereas RYGB surgery resulted in increased levels of Veillonella, Slackia, Granucatiella and Acidaminococcus. Furthermore, among SG subjects an increase in the abundance of Lactobacillales [68,69] and on species level in Bacteroides uniformis [70,71] and Roseburia intestinalis, and a decrease in the phylum Firmicutes [72,73], the family Bifidobacteriaceae [63,73] and the species Coprococcus comes [71,72] and Dorea longicatena [71,72] has been observed.

Some taxonomic changes following RYGB surgery have been associated with the resolution of comorbidities and weight loss. High levels of Gammaproteobacteria phylotypes and Lactobacillus have been associated with weight loss after RYGB surgery [65]. Furthermore, a negative correlation between blood glucose levels and Lactobacillus abundance has been found. This association remained significant even after correction for calorie intake [58]. An increase in F. prausnitzii has been observed after RYGB surgery and was shown to be inversely correlated with inflammatory markers [52]. However, this correlation was dependent on calorie intake. In this study, the microbial adaptation following RYGB surgery could be due to energy restriction. On the contrary, Graessler et al. observed decreased levels of F. prausnitzii, which were correlated with a reduction in C-reactive protein [55].

However, the microbial changes observed following gastric bypass are not consistent between studies (Table 1). This could be caused by differences in the techniques used for microbiome analysis (e.g., pyrosequencing, metagenomic, RT-qPCR), different time frames of follow up post-surgery, pre-surgical presence of T2DM and medication (e.g., proton-pump inhibitors, statins or antidiabetic drugs) used in the cohorts. One of the weak points in some studies is that the comparison of gut microbiota pre- and post-surgery is missing (Table 1). Considering the inter-individual variation in the gut microbiota composition, using obese subjects as controls does not entirely represent preoperative microbiota. Furthermore, results could be biased by differences in food intake, reduced digestion and changes in food choices and preferences. It is well documented that diet is an important factor shaping the composition and function of intestinal microbiota. Following gastric bypass surgery, dietary intake is altered both in quantity and quality. Bariatric surgery leads to a 40%–50% reduction in energy intake in the first six months post-surgery. Compared to pre-surgery values, energy intake is decreased by 1215 kcal/d after RYGB surgery [81]. Studies have indicated an improvement in food intake with a reduced fat and carbohydrate intake in the early postoperative period [82]. After the first year, food intake tended to return to pre-surgery habits [82,83].

In many bariatric surgical centres, a very low-calorie diet (VLCD) is regularly prescribed before surgery. Excess body fat is known to exacerbate the technical aspects of surgery increasing both the operating time and the risk of complications [28]. Thus, it could be assumed that at the time of baseline faecal sampling, the majority of patients already had been on a VLCD resulting in atypical microbial compositions and, therefore, an incorrect baseline. To the best of our knowledge, there is only one study considering restrictive diets prior to bariatric surgery [61]. Moreover, several of the studies we reviewed did not assess food intake post-surgery or adjust for changes in food intake and digestion after data collection. Thus, whether the observed alterations in gut microbiota are a direct consequence of surgery itself or of the alterations in food intake and/or weight loss still remains unclear. For this purpose, mouse models, using calorie restriction or sham-operated pair-fed individuals as diet-matched controls could be a helpful tool. There is some evidence from experimental studies on rats, which indicates that the observed effects on gut microbiota composition and diversity after RYGB surgery are associated with surgery itself [84,85]. Guo et al. found similar glucose improvement and increased gut microbiota diversity 10 weeks after RYGB and SG surgery compared with the sham-operated groups (pair-fed as RYGB or fed *ad litidum*). The presence of the pair-fed group as an RYGB diet-matched control group indicated that the increased diversity is caused by RYGB surgery and consequently may be independent of food intake. Following RYGB surgery a higher relative abundance of Proteobacteria/Gammaproteobacteria and Betaproteobacteria and an increase in levels of Fusobacteria and Clostridium compared to SG and sham-operated groups was observed, while SG caused an increase in the relative abundance of Actinobacteria compared with the other groups. Most of the 12 discriminate microbial genera that were affected by RYGB surgery, correlated with alterations in metabolic phenotype. However, only 28.6% of these correlations remained significant after adjustment for body weight and four discriminant genera negatively correlated with serum insulin level independent of food intake and weight loss after RYGB surgery [84].

In addition to the microbial changes after bariatric surgery, Paganelli et al. also investigated short-term alterations in the microbiome following a VLCD prior to either RYGB or SG surgery. They found that such a crash diet (500 kcal/d) induced profound but temporary changes in the gut microbiome diversity and composition. Contrary to the crash diet, surgery was associated with the early and persistent replacement of distinct bacterial taxa and restoration of the gut microbial diversity. The VLCD resulted in a temporal increase in the relative abundance of Bifidobacterium and a decrease in the relative abundance of Streptococcaceae, whereas the opposite effect was observed after surgery. These alterations persisted for at least six months. With regard to relative abundance and beta-diversity of enteric bacteria, there were no differences between patients undergoing either RYGB or SG surgery: neither prior to surgery nor at any time point afterwards. In addition, the weight loss in patients was comparable between the two surgeries. Hence, the authors concluded that the comparable weight loss could be causative for the similar changes in gut microbiota composition observed after both surgery types. In this study, bariatric surgery itself, unlike a VLCD, resulted in marked and sustained alterations of the gut microbiota composition [61]. Murphy et al. examined enteric microbial changes after RYGB or SG surgery in obese patients with T2DM. At the time of baseline faecal sampling, all patients had been on a VLCD using a formula diet (Optifast, 152–207 kcal per serving) plus green, non-starchy vegetables daily for at least two weeks. RYGB surgery resulted in an increase in the gut microbiota diversity, which was not observed after SG. Three major phyla changes with increased Firmicutes and Actinobacteria and decreased Bacteroidetes phyla were detected after RYGB surgery. Contrarily, SG surgery caused an increase in Bacteroidetes phyla. An increase in Roseburia species was associated with diabetes remission for both types of surgery [59].

Given the known association between food intake and gut microbial richness and the dramatic changes in food intake after gastric bypass surgery, Al Assal et al. recently investigated the microbial profile from obese diabetic women before and after RYGB surgery while taking into account the nutritional impact. Therefore, the authors evaluated the microbiome from 25 patients before, three months after and 12 months after surgery. Food intake was calculated using a seven-day food record. RYGB surgery resulted in changes in the relative abundance of some gut bacteria genera (Table 1), increased gut microbial richness and induced a decrease in the Firmicutes to Bacteroidetes ratio 12 months post-RYGB surgery. Microbial richness level was correlated with diet composition both in the pre- and post-surgery state. However, increased microbial richness was not associated with total diabetes remission. The genus richness had a positive correlation with the fibre/lipid intake ratio and the fibre intake alone and it also had a negative correlation with the lipid intake both pre and postoperatively. Specifically, a direct correlation was exhibited between the lipid intake and levels of one unclassified genus of Acidaminococcaceae and an inverse correlation was exhibited between the lipid intake and Parabacteroides levels. Protein intake had an inverse correlation with Akkermansia levels and a direct correlation with levels of one unclassified Veillonellaceae. Interestingly, only patients achieving diabetes remission increased their total soluble and insoluble fibre intake and decreased saturated fat intake [80]. Thus, additionally to bariatric surgery-induced microbial adaptation, the diet itself, on a macronutrient level, affects the microbial composition and consequently weight loss.

## 5. Diet and Microbiome

As previously stated, diet plays a key role in shaping the microbiome. Alterations in dietary habits can induce microbial shifts within 24 h, which are determined by the competition for substrates and the toleration of gut conditions [15,86]. The strongest dietary influence on the formation of the microbial metabolites is exerted by non-digestible carbohydrates, protein and fat [86]. In studies with digestible carbohydrates, where humans were fed high levels of glucose, fructose and sucrose, an increased relative abundance of Bifidobacteria and a reduced count of Bacteroides was observed. Non-digestible carbohydrates, however, seem to have a positive effect on microbiota gene richness. A diet rich in non-digestible carbohydrates increases the abundance of intestinal Bifidobacteria and lactic acid bacteria [15]. Bifidobacteria are beneficial bacteria in the gut whose growth can be stimulated with prebiotic fibre ingestion [87]. Prebiotics, which are defined as non-digestible dietary components that benefit their host’s health via the selective stimulation of growth and/ or activity of certain microorganisms, induce shifts in the gut microbiome [15]. Major products of the fermentation of carbohydrates, such as the short-chain fatty acids propionate and butyrate, serve as an energy source for the host and provide anti-inflammatory and anti-apoptotic effects [86]. The Prevotella enterotype, which is one of the leading causes of inter-individual gut microbiota variations, is associated with high values of carbohydrates and simple sugars in the diet. It is hypothesised to be sensitive to long-term fibre intake [88,89]. Vegetarians and members of agrarian societies show enrichment in Prevotella [88,90].

Protein consumption positively correlates with an overall microbial diversity. Mainly the counts of bile-tolerant anaerobes, such as Bacteroides, Alistipes and Bilophila, increase after the consumption of animal-based protein [15,91]. At the same time some bacteria that are generally regarded as a beneficial decrease in their abundance on a high protein diet, including the butyrate producer F. prausnitzii, Ruminococcus and the mucin degrading Akkermansia [91]. The effect also depends on the source of protein, for example, an increased dietary protein content through the consumption of red meat is associated with an increased formation of toxic bacterial metabolites that create a less favourable gut environment [86]. Plant-derived proteins seem to be more favourable for promoting beneficial microbiota that have positive effects on the host’s metabolism [91], which could be related to the protein quality itself or the indirect increase of fibre as a side effect of the consumption of plant-derived proteins. Whereas an animal-based diet not only increases the bile-tolerant microorganisms but at the same time reduces the abundance of Firmicutes, such as Roseburia, Eubacterium rectale and Ruminococcus bromii, which preferentially metabolise dietary plant polysaccharides [89]. As observed for an animal-based protein diet, a high-fat diet also increases the total anaerobic microflora as well as the abundance of Bacteroides [15]. A changed microbiota induced by a high-fat diet can even trigger metabolic inflammation through a greater gut permeability allowing Lipopolysaccharides to enter systemic circulation [92].

The Western diet, which as a dietary pattern is typically high in animal protein, sugar, starch, fat and low in fibre, combines these different aspects discussed above and leads to a distinct decrease in numbers of both total bacteria and beneficial Bifidobacterium and Eubacterium species [14,90]. The cecal community of individuals consuming Western-associated diets contain a higher relative abundance of Firmicutes and a lower relative abundance of Bacteroidetes [23]. This was highlighted in a study by De Filippo et al. which compared the gut microbiome of children from Europe (Florence, Italy) and rural Africa (Burkina Faso). The diet from children in Burkina Faso was low in fat and animal protein but rich in starch, fibre and plant polysaccharides and, therefore, resulted in a significant enrichment in Bacteroidetes and a depletion in Firmicutes. A unique abundance of Prevotella and Xylanibacter was found. This allows for the supposition that the exposure to a large variety of environmental microbes, which is associated with the high-fibre diet of agrarian societies, could have a positive effect on potentially beneficial bacterial genomes. The European children, on the other hand, consumed a typical Western diet. They showed an increased Firmicutes/Bacteroidetes ratio probably induced by the high-calorie diet. However, total microbial richness and biodiversity was lower in the European as opposed to the African children [90].

In addition to the studies on high-caloric diets, there are also studies about VLCD and their influence on the gut microbiome. Louis et al. managed to detect some variations at the genera and species level but none at the phyla level after consumption of the VLCD in the form of formula (OPTIFAST ^®^ Nestlé, 800 kcal/d) over a period of three months. The formula was enriched with inulin to improve bowel movement. Despite the supplementation with inulin, butyrate-producing Roseburia decreased during the phase of the intervention. Other than the response of the microbiota to the dietetic and lifestyle intervention, there were no significant changes observed and the initial gut microbiome was restored at a taxonomical and functional level one year after the very low-calorie diet. The only exception was an increase in the abundance of Akkermansia which persisted throughout the study [93]. Several other studies showed similar outcomes when using the same VLCD formula. These other studies also reported alterations in the gut microbial diversity and bacterial metabolism during the three months of intervention phase but the VLCD-induced changes diminished after ending the intervention and went back to baseline levels during the weight maintenance phase [94]. Furthermore, the energy restriction did not affect alpha-diversity and did not trigger consistent shifts in the phylogenetic composition between individuals [95]. Only one study by Damms-Machado et al. demonstrated a change in the Bacteroidetes/Firmicutes ratio after 12 weeks of a VLCD programme. The dietary intervention resulted in a reduced abundance of Bacteroidetes in favour of Firmicutes. This rise is mainly due to butyrate-producing bacterial strains that can process the inulin-enriched formula. Despite the overall growth in Firmicutes, which is typically associated with an increased energy harvest for the host, the specific growth of butyrate-producing species may result in positive metabolic effects [72].

In addition to its influence on microbial composition, preoperative weight loss with a VLCD can help simplify gastric surgeries [29]. In particular, an enlarged liver compromises the visibility of the gastroesophageal area making it more friable and, therefore, more prone to bleeding [26]. In a study by Lewis et al., after six weeks of an Optifast^®^ diet, the mean liver volume was reduced by 14.7% and the mean liver fat by 43%. This suggests that the reduction in liver volume is due to a loss of fat [96]. Similar results can be found in a clinical trial by Gils Contreras et al. where the liver volume was reduced by 15.6% after three weeks of VLCD. The reduction was directly related to the baseline BMI [26]. Generally, the highest decrease in liver volume seems to be achieved in the first two weeks of the diet [28,29]. Although a reduced liver size might positively influence the surgeon’s perceived difficulty of the procedure, there were no differences found regarding mean operating time, estimated blood loss or intraoperative complications compared to control groups without preoperative VLCD [28,97]. Only the risk of postoperative complications, especially infections, decreased after the preoperative diet in comparison to no prior diet [28]. However, most studies do not assess the diet-induced microbial alterations prior to surgery resulting in a biased comparison of the atypical microbial compositions at baseline and after surgery.

## 6. Conclusions

Even though bariatric surgery is established as the only effective treatment option for achieving sustained weight loss and metabolic improvement, the exact mode of action is still unresolved. The total extent of weight loss exhibits high inter-individual variation in patients (responder vs non-responders). Our research shows that it is likely that differential changes in gut microbiota account for the variability found after gastric bypass surgeries. In addition to the surgically induced microbial adaptation, the diet itself, on a macronutrient level, affects the microbial composition and, therefore, weight loss. It is well documented that diet is an important factor shaping the composition and function of intestinal microbiota. However, most studies on bariatric surgery do not assess food intake pre- or post-surgery or adjust for changes in food intake and digestion afterwards. Thus, further research efforts are needed to deepen the understanding of gut microbial changes after bariatric surgery, which induce weight loss and metabolic improvements, and the relative impact of nutrition.

## Figures and Tables

**Table 1:** Changes in the gut microbiota following bariatric surgery in humans.

Reference	Subjects	Type of Surgery (*n*)	Sample Size (*n*)	Time Points	Pre-BS Dietary Intake	Post-BS Dietary Intake	Impact on Diversity and Gene Richness	Changes in Relative Abundance			
								Phylum	Class/Order/Family	Genus	Species
Zhang 2009 [67]	Normal weight, obese, post-BS	RYGB	6 RYGB 3 NW 3 MO 3	8–15 mo post-BS	-	-	-	↑ Verrucomicrobia ↑ Fusobacteria ↓ Firmicutes	↑ Gammaproteobacteria ↑ Prevotellaceae ↑ Fusobacteriaceae ↑ Enterobacteriaceae ↓ Clostridia	↓ Lachnospira ↑ Akkermansia	
Furet 2010 [52]	Post-BS (7 T2DM), lean controls	RYGB	43 RYGB 30 NW 13	Pre-BS, 3, 6 mo post-BS	1-h questioning period	1-h questioning period	-	↑ Bacteroidetes		↑ Bacteroides/Prevotella ratio ↓ Bifidobacterium ↓ Lactobacillus ↓ Leuconostoc ↓ Pediococcus	↑ Escherichia coli ↑ Faecalibacterium prausnitzii
Kong 2013 [65]	Morbidly obese women	RYGB	30	Pre- BS, 3, 6 mo post-bs	1-h questioning period	1-h questioning period	↑ GM richness	↑ Proteobacteria ↓ Firmicutes		↑ Alistipes ↑ Escherichia ↑ Bacteroides ↓ Bifidobacterium ↓ Lactobacillus ↓ Dorea ↓ Blautia	
Graessler 2013 [55]	Morbidly obese subjects	RYGB	6	Pre- BS, 3 mo post-bs	-	-	-	↑ Proteobacteria ↑ Fusobacteria ↑ Verrucomicrobia ↓ Bacteroidetes ↓ Firmicutes ↓ Actinobacteria ↓ Cyanobacteria ↑ Bacteroidetes/Firmicutes ratio		↑ Enterobacter ↑ Neurospora ↑ Citrobacter ↑ Veillonella ↑ Salmonella ↓ Faecalibacterium ↓ Coprococcus ↓ Helicobacter ↓ Anaerostipes ↓ Nakamurella	↑ Enterobacter cancerogenus ↑ Veillonella parvula ↑ Veillonella dispar ↑ Shigella boydii ↑ Salmonella enerica ↓ Lactobacillus reuteri ↓ Treponema pallidum ↓ Mycobacterium kansasii ↓ Faecalibacterium prausnitzii ↓ Clostridium comes
Ward 2014 [74]	Severely obese subjects	RYGB	8	Pre- BS, 6 mo post-bs	-	-	-	PPI Users: ↑ Bacteroidetes ↑ Proteobacteria PPI non-users: ↓ Verrucomicrobia ↓ Firmicutes ↓ Proteobacteria			
Tremaroli 2015 [56]	Post-BS women, non-operated severely obese women	RYGB VGB	21 RYGB 7 VGB 7 MO 7	9.4 y post-BS	-	-	-	↑ Proteobacteria ↓ Firmicutes	↑ Gammaproteobacteria		↑ Escherichia coli ↓ Clostridium difficile ↓ Clostridium hiranonis ↓ Gemella sanguinis
Federico 2016 [75]	Severely obese and normal weight	BIB	56 BIB 28 NW 28	Pre- BS, 6 mo post-bs	7 d food records	7 d food records	-				↑ Lactobacillus crispatus ↑ Streptococcus spp. ↑ Megasphaera sp.
Palleja 2016 [57]	Morbidly obese subjects	RYGB	13	Pre- BS, 3, 12 mo post-bs	Weight loss diet (8% weight loss)	-	↑ species richness ↑ gene richness	↑ Proteobacteria ↑ Fusobacteria			↑ Escherichia coli ↑ Klebsiella pneumonia ↑ Akkermansia muciniphila ↓ Faecalibacterium prausnitzii ↓ Anaerotruncus colihominis ↓ Megasphaeara micronuciformis ↑ Alistipes spp. ↑ Streptococcus spp. ↑ Veillonella spp.
Patrone 2016 [58]	Severely obese	BIB	11	Pre- BS, 6 mo post-bs	Assessment of dietary habits	Assessment of dietary habits	↓ Species richness			↓ Lachnospiraceae ↓ Clostridiaceae ↓ Ruminococca-ceae ↓ Eubacteriaceae ↓ Coriobacteriaceae ↑ Lactobacillus ↑ Megasphaera ↑ Acidaminococcus	
Ilhan 2017 [54]	Pre-BS obese, normal weight, post-RYGB and post-LAGB	RYGB LABG	63 RYGB 24 LAGB 14 NW 10 Preb-Ob 15	35 ± 8 mo post-BS		4 d food diaries and FFQ	↑ α-diversity		↑ Gammaproteobacteria ↑ Bacilli ↑ Flavobacteria ↑ Fusobacteria	↑ Escherichia ↑ Veillonella ↑ Streptococcus ↑ Trabulsiella ↑ Haemophilus ↑ Coprococcus ↑ Enterococcus ↓ Oscillospira ↓ Coprobacillus ↓ Bacteroides	
Murphy 2017 [59]	Obese T2DM subjects	RYGB SG	14 RYGB 7 SG 7	1 w pre-BS, 1 y post-BS	2 w Optifast 3 d food diary	3 d food diary	↑ α-diversity	↑ Firmicutes ↑ Actinobacteria ↓ Bacteroidetes			
Aron-Wisnewsky 2018 [60]	Severely obese subjects	RYGB agb	61 RYGB 41 Agb 20	Pre- BS, 1, 3, 12 mo post-bs	Equilibrate diet	-	↑ Microbial gene richness	↑ GU:99 Roseburia ↑ GU:225 Butyricimonas virosa ↑ GU:359 Butyricimonas			
Campisciano 2018 [70]	Obese patients, normal weight controls	LGB SG	40 Sg 10 LGB 10 NW 20	Pre- BS, 3 mo post-bs	-	-	↑ α-diversity	↑ Proteobacteria ↑ Firmicutes ↓ Actinobacteria ↓ Bacteroidetes ↑ Firmicutes/Bacteroidetes ratio		↑ Prevotella/bacteroides ratio ↑ Prevotella ↓ Bacteroides	↑ Bifidobacterium vulgatus ↑ Hafnia alvei ↓ Bifidobacterium uniformis
Cortez 2018 [76]	Overweight, class I or II obesity T2DM patients, medical care	DJB	21 DJB 11 SC 10	Pre-BS, 6, 12 mo post-BS	-	SC: diet formulated using total energy expenditure	↓ α-diversity	↑ Bacteroidetes ↑ Verrucomicrobia		↑ Bacteroides ↑ Akkermansia ↑ Dialister	↑ Akkermansia muciniphila
Paganelli 2019 [61]	Morbidly obese	RYGB SG	45 Sg 22 RYGB 23	Before VLCD, 2 w after VLCD, 1 w, 3, 6 mo post-bs	2 w modifast (500 kcal/d)	-	Post-VLCD: ↓ α-diversity 3 and 6 mo: ↑ α-diversity to baseline level		Post-VLCD: ↑ Rikenellaceae ↓ Streptococcaceae ↓ Ruminococcaceae post-BS: ↑ Streptococcaceae ↑ Enterobacteriaceae ↓ Bifidobacteriaceae		
Sanchez-Alcoholado 2019 [63]	Severely obese patients	RYGB SG	28 RYGB 14 SG 14	Pre- BS, 3 mo post-bs	-	-	~α-diversity	↑ Proteobacteria ↑ Fusobacteria	↑ Fusobacteriaceae ↑ Clostridiaceae ↑ Enterobacteriaceae ↓ Bifidobacteriaceae ↓ Peptostrepto-coccaceae	↓ Bifidobacterium ↓ Collinsella ↑ Slackia ↑ Clostridium ↑ Veillonella ↑ Granucatiella ↑ Oscillospira ↑ Fusobacterium ↑ Granucatiella	
Pajecki 2019 [77]	Super-obese subjects	RYGB	9	Pre- BS, 12, 24 mo post-bs	-	-		↓ Proteobacteria			
Lee 2019 [78]	Mildly or moderately obesity with T2DM at 10% of weight loss	RYGB AGB	12 AGB 4 RYGB 4 MWL 4	Pre-BS, at 10% of weight loss, 9 mo if 10% was not achieved	-	-	↑ α-diversity ↑ richness	↑ Proteobacteria ↑ Actinobacteria		↑ Faecalibacterium ↑ Akkermansia	
Fouladi 2019 [53]	Post-RYGB with successful or poor weight loss, non-surgical controls	RYGB	18 SWL 6 PWL 6 NSC 6	2–5 years post-BS	-.	24-h recall for 3 days	↑ α-diversity ↑ richness PWL vs. NW ↑ diversity		↑ Micrococcales ↑ Lactobacillales	↑ Rothia ↑ Streptococcus PWL vs. NW: ↑ Oscullibacter ↑ Lactobacillus ↑ Enterobacter ↑ Akkermansia	
Gutierrez-Repiso 2019 [44]	Post-RYGB with primary failure, weight regain or successful weight loss	RYGB	24 SWL 6 Primary failure 6 Weight regain 12	8.3 ± 1.7 years post-BS	-	-	~α-diversity			Success vs. weight regain: ↑ Butyrivibrio ↑ Lachnospira ↑ 5–7N15 ↑ Sacina ↑ Alkaliphilus ↑ Pseudo-altermonas ↑ Cetobacterium ↑ AF12	
Palmisano 2019 [64]	Obese patients, normal weight controls	RYGB SG	50 RYGB 9 Sg 16 NW 25	Pre- BS, 3, 6 mo post-bs	Food preferences	Food preferences	~α-diversity	↑ Proteobacteria ↑ Fusobacteria ↑ Verrucomicrobia ↓ Bacteroidetes ↓ Firmicutes	↑ Gammaproteobacteria		6 mo: ↑ Akkermansia muciniphila ↓ Veillonella atypical ↓ Veillonella dispar ↓ Streptococcus gordonii ↓ Streptococcus australis ↑ Yokenella regensburgei ↑ Fusobacterium varium
Shen 2019 [79]	Severely obese with and without T2DM	RYGB SG	26 RYGB 19 SG 7	Pre-BS, 3, 6, 12 mo post-BS	-	-	6 mo: ↑ for α-diversity ↑ β-diversity 12 mo: Tend to pre-BS levels	3 and 6 mo: ↑ Verrucomicrobia ↑ Proteobacteria 12 mo: trend diminished		↑ Akkermansia	
Al Assal 2020 [80]	Obese T2DM women	RYGB	24	Pre-BS, 3, 12 mo post-BS	7 d records (1700 kcal/d)	7 d records	↑ GM richness	3 mo: ↑ Proteobacteria ↑ Firmicutes ↑ Actinobacteria 12 mo: ↓ Firmicutes/bacteroidetes ratio		3 mo: ↑ Veillonella ↑ Streptococcus ↑ Gemella ↑ Oribacterium ↑ Atopobium ↑ one unclassified Lactobacillus genus ↑ Leptotrichia ↑ Neisseria ↑ one unclassified Pasteurellaceae genus ↓ Faecalibacterium	

↑—increase, ↓—decrease, ~—unchanged, ↑ tended to increase, AGB—adjustable gastric banding, BIB—biliointestinal bypass, BS—bariatric surgery, DJB—duodenal–jejunal bypass, FFQ—food frequency questionnaire, GM—gut microbial richness, LAGB—laparoscopic adjustable gastric banding, LGB—laparoscopic gastric bypass, MO—morbidly obese, MWL—medical weight loss, NSC—non-surgical control, PPI—proton-pump inhibitor, PWL—poor weight loss, RYGB—Roux-en-Y gastric bypass, SC—standard care group, SG—sleeve gastrectomy, SWL—successful weight loss, T2DM—type 2 diabetes mellitus, VGB—vertical banded gastroplasty, VLCD—very-low-calorie diet.
